# A feasibility study on the efficacy of a VR-based mindfulness intervention for dementia caregivers in the home environment: A pilot randomized control trial protocol

**DOI:** 10.1371/journal.pone.0347988

**Published:** 2026-04-29

**Authors:** Qi Wang, Doris Yu, Gloria Wong, Daisy Zhang, Martin Knapp, Padmore Adusei Amoah, Jia Li, Cheng Shi

**Affiliations:** 1 Centre on Behavioral Health within the Faculty of Social Sciences, The University of Hong Kong, Hong Kong SAR, China; 2 School of Nursing, The University of Hong Kong, Hong Kong SAR, China; 3 School of Psychology and Clinical Language Sciences, University of Reading, Reading, United Kingdom; 4 School of Nursing, The Hong Kong Polytechnic University, Hong Kong SAR, China; 5 Department of Health Policy, The London School of Economics and Political Science, London, United Kingdom; 6 School of Graduate Studies, Lingnan University, Hong Kong SAR, China; 7 Department of Social Work, The Chinese University of Hong Kong, Hong Kong SAR, China; Arab American University, PALESTINE, STATE OF

## Abstract

**Background:**

Dementia affects over 50 million people worldwide, with numbers projected to triple by 2050. Informal caregivers—predominantly family members—provide the majority of daily care and exhibit disproportionately high rates of psychological distress, depressive symptoms, and perceived burden. Although mindfulness-based interventions (MBIs) have consistently demonstrated moderate-to-large effect sizes for improving caregiver mental health, traditional delivery modalities—face-to-face sessions or audio-guided home practice—are frequently constrained by low adherence, scheduling conflicts, and limited ecological validity within domestic settings. Immersive virtual reality (VR) technology offers a novel platform that can simulate restorative natural environments, minimise external distractions, and standardise mindfulness instructions while preserving temporal flexibility for users. Emerging evidence from non-caregiver populations suggests that VR-based MBIs may enhance engagement, retention, and affective outcomes compared with audio-only equivalents; however, data specific to dementia caregivers remain absent. This pilot study aims to explore the feasibility, acceptability, and effectiveness of a VR-based MBI in improving psychological status and caregiver-recipient relationship dementia caregivers.

**Methods:**

This is a 3-arm, parallel, single-blinded, pilot randomised control trial comparing VR-based MBI with audio-based MBI and care-as-usual. The target sample includes 90 caregivers of community-dwelling persons with dementia. Each arm (n = 30) will receive an eight-week exercise with instructions, with outcome assessment at baseline, post-treatment, and a 2-month follow-up. Qualitative interviews will also be conducted to assess the feasibility and acceptance. The VR-based MBI group will engage in mindfulness exercises using a mobile app and VR technology, incorporating different natural environments. The primary outcome is the caregivers’ psychological status regarding depression, anxiety, and stress. The secondary outcomes include caregiver burden, mindfulness level, quality of life and caregiver-recipient relationship. Following intention-to-treat analysis, quantitative data on effectiveness will be analysed using between-group t-tests and group-by-time effect size (Cohen’s d). A six-step thematic analysis will be utilized for qualitative data.

**Conclusion:**

The proposed intervention is expected to improve the psychological status of caregivers of people with dementia. The research may be helpful in promoting the well-being of people suffering from other psychological issues in the future.

**Trial registration:**

ClinicalTrials.gov NCT07103434

## Introduction

In Hong Kong, around 15.5% of dementia caregivers experience burnout [1] and this was especially during the COVID-19 pandemic when 64.7% of dementia caregivers reported suffering from probable depression and increased levels of anxiety and stress [2]. The Chief Executive’s 2022 Policy Address highlights enhancing support for caregivers and enhancing their mental well-being. However, very limited support is currently available for caregivers of people with dementia in the local community. There is an urgent need for a self-instructed, flexible, and convenient intervention within the home environment to effectively enhance caregivers’ psychological well-being in Hong Kong. This project will develop a new type of mindfulness-based intervention (MBI) using virtual reality (VR) to reduce caregivers’ psychological distress. We anticipate that this project will increase societal awareness of the burden borne by caregivers of people with dementia.

Mindfulness refers to “the awareness that emerges through paying attention on purpose, in the present moment, and non-judgmentally to the unfolding of experience moment by moment” [3]. Several recent systematic reviews provide robust evidence for the medium to large effect of MBIs on reducing caregivers’ psychological distress, depression, burden, and enhancing quality of life by improving their ability to cater to the needs of people with dementia and managing daily stress and emotional challenges associated with caregiving [4,5], as well as enhancing relationship between caregivers and recipients [4].

Dementia caregivers bear significant daily caregiving responsibilities and dedicate the majority of their time to companionship and ensuring the safety of people with dementia. This responsibility often leaves caregivers with limited time and availability to engage in mindfulness practice outside of the home. However, the practice of mindfulness requires conscious effort and can be difficult to maintain in the home environment, especially for novice meditators who are already expending cognitive resources to control their self-regulatory skills [6]. Beginning mindfulness practitioners commonly report easily being distracted during practice, fall asleep, wrestle with tiredness or ambivalence, difficulty of incorporating mindfulness practice into their daily life [7]. The adoption of digital technology may support beginners’ mindfulness practice, such as Web-based interventions [8] or smartphone apps [9] that can deliver guided audio- recorded mindfulness practice in facing the challenges of practicing and adhering to mindfulness [10]. However, there are limitations with these audio-based MBIs, including a low adherence rate and the failure of participants to maintain these practices over the long term [10].

VR-based mindfulness practice in a pleasant and immersive virtual environment may relieve physical and mental discomforts frequently experienced by beginning mindfulness practitioners without compromising their present-moment awareness [11]. It also provides access to physically or cognitively inaccessible stimuli, enhancing guided imaginary mindfulness [12]. Furthermore, VR also facilitates participants’ application of mindfulness skills to real-life practice, and its non- traditional format may further increase their acceptance of the therapy [11].

VR-based MBI has recently been proposed as a medium to support mindfulness practice and become an effective way to improve participants’ psychological well-being and cultivate spiritual experiences, especially strong feelings of awe [13]. Specifically, compared to audio-based mindfulness, VR-based MBIs have been shown to offer several additional benefits, including improved well-being [14], higher treatment adherence [15], reductions in vigor, fatigue, and confusion [16] and more enjoyable experiences [17].

However, VR-based mindfulness practice studies are predominantly conducted in a clinic environment or specific settings rather than a home environment [11]. Practicing in home environment may enable participants to feel safe and remove their concerns about leaving the person with dementia. VR-based MBI practice can be time- and cost-efficient, simple to initiate and complete, and practical for caregivers at home.

Meanwhile, local evidence on VR-based interventions targeting psychological health issues such as depression, anxiety, and stress [18], alongside studies on the benefits of mindfulness for dementia caregivers’ well-being [19] provides form a foundation for contemplating the integration of VR technology into mindfulness training programs.

### Research hypotheses

The research hypotheses of this proposed study include the following:

1 Primary Outcome

Participants in the VR-based mindfulness-based intervention (MBI) group will demonstrate greater improvements in psychological status compared to those in the audio-based MBI group, and participants in the audio-based MBI group will demonstrate greater improvements than those in the waitlist control group.

2 Secondary Outcomes

a. Participants in the VR-based MBI group will demonstrate greater improvements in caregiver burden, mindfulness level, and quality of life compared to those in the audio-based MBI group, with the audio-based MBI group showing greater improvements than the waitlist control group.

b. Caregivers in the VR-based MBI group will show greater improvements in caregiver-care recipient relationship quality compared to the audio-based MBI group, with the audio-based MBI group showing greater improvements than the waitlist control group.

## Methods

### Trial design

**Fig 1:**
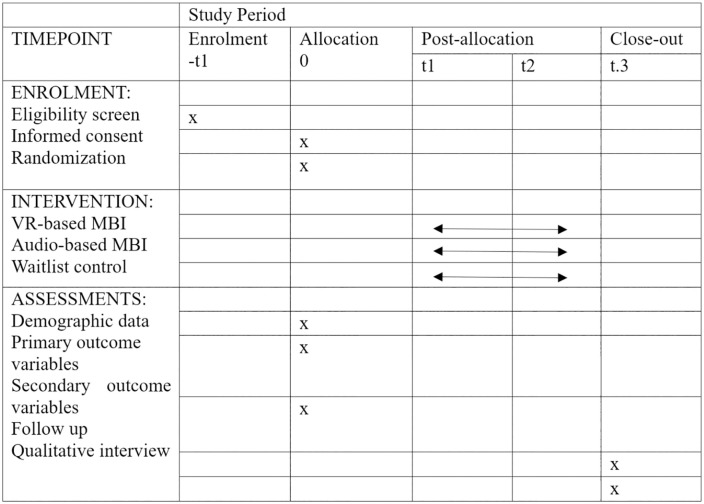
SPIRIT schedule of enrolment, intervention and assessment. Notes: -t1, 0, baseline; t1, start of the intervention; t2, 8 weeks after the start of the intervention; t3: 2-month follow-up.

**Fig 2:**
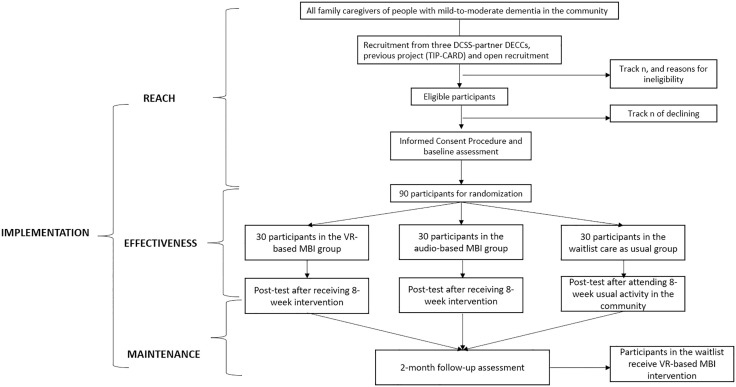
Consort diagram of procedures for randomised controlled trail design and RE-AIM considerations.

This proposed study will be a pilot RCT to examine the feasibility, acceptability and potential effectiveness of implementing the intervention with caregivers of people with dementia. Ethics approval has been obtained from the Office of Research and Knowledge Transfer, Lingnan University (LU Ethics: EC021–2425) and Institutional Review Board of the University of Hong Kong/ Hospital Authority Hong Kong West Cluster (HA HKW IRB: UW24–706). The SPIRIT schedule of enrolment, interventions, and assessments can be found in Fig 1 (The SPIRIT checklist can be found in supplementary materials). The flowchart of the design based on RE-AIM framework [20] can be found in Fig 2.

### Study setting

This study will be conducted among family caregivers of community-dwelling people with dementia at their home environment in Hong Kong. The specific study sites will be the current living environment of the family caregivers for their convenience.

### Participants

The participants will comprise family caregivers of persons with mild-to-moderate dementia. Compared to caregivers of severe dementia (who may be too overwhelmed) or preclinical stages (who may not yet recognize the need), mild-to-moderate dementia caregivers can be actively managing daily care challenges (e.g., behavioral symptoms, medication adherence) and more likely to retain and apply intervention strategies due to relatively lower stress levels than in advanced stages.

The inclusion criteria will comprise: (1) Aged 18 years or older;(2) Primary family caregivers who spend over four hours/day taking care of a community-dwelling person who has been clinically diagnosed with any type of mild-to-moderate dementia at least 6 months; (3) Self-reported psychological distress, operationally defined as answering “Yes” to the screening question: “Are you currently experiencing psychological distress?”; and (4) Able to speak and read Cantonese. The exclusion criteria included: (1) Have practiced mindfulness-based intervention for at least 3 months in the past; (2) Have hearing or visual impairment that cannot practice mindfulness via wearing VR glasses embedded in a mobile phone app; (3) Do not own a smart phone that can install the VR-based MBI app; (4) Caring for a person with dementia living in a residential care setting; (5) Caring a person with severe level of dementia; (6) Have been diagnosed with any mental disorder or on-site of psychotic disorders; (7) Receiving any other type of mental health intervention; (8) Participants with a history of motion sickness (due to the risk of VR triggering motion sickness); and (9) Unable to understand Cantonese. Exclusion of individuals with current mental disorders is intended to ensure participant safety and because the intervention is not designed to provide clinical treatment for psychiatric conditions. Participants who screen positive for severe symptoms will be provided with information on appropriate support services. Moreover, eligibility requirements related to smartphone compatibility and absence of motion sickness may preferentially include caregivers who are younger, healthier, or more technologically confident. These factors may limit generalizability to the broader population of dementia caregivers and will be considered when interpreting study findings and planning future trials.

### Sample size

This pilot three-arm randomized controlled trial will enrol 90 participants (30 per arm). The primary purpose of this sample size is to evaluate feasibility (recruitment, retention, adherence, and data completeness) and to obtain preliminary estimates of outcome variability and treatment effects to inform a fully powered trial. Previous studies in comparable populations have reported standardized effect sizes in the range of 0.40–0.54 [13,15]. With 30 participants per group, the present pilot may not have enough statistical power to formally test these effects but allows estimation of group differences and their variability with sufficient precision to guide sample size calculations for a future definitive trial. For example, this sample size is expected to yield confidence intervals around standardized effect estimates that are sufficiently narrow to distinguish between small and moderate effects and to support planning of the definitive trial. This sample size also ensures adequate representation across study arms for process evaluation and qualitative feedback through focus group discussions while remaining feasible within available resources.

### Recruitment

First, we will recruit our participants through the District Elderly Community Centers (DECCs) participating in the Dementia Community Support Scheme (DCSS). The Hong Kong SAR Government has launched the DCSS to provide multi-disciplinary community support services through medical-social collaboration for people with mild or moderate dementia and family carers since 2017 (The Government of the Hong Kong SAR, 2018, July 4). Three DECCs in the Tuen Mun area, who are DCSS partners, will serve as the main recruitment sites for the proposed study. Around 330 caregivers of people with dementia have been registered as DCSS service users within these three DECCs by the middle of 2023.

Second, building upon prior engagements with dementia caregivers, such as past participants, we will also employ targeted outreach methods. We will send mass email or short message to participants enrolled in the team’s previous projects, such as a study on dementia, Tools to information Policy: Chinese Communities’ Action in Response to Dementia (TIP-CARD, https://tip-card.hku.hk/), where 750 dyed people with dementia and their family caregivers of the survey in 2021–2022 have already consented to being contacted for other dementia-related projects.

When necessary, we will also conduct open recruitment. We will distribute our recruitment poster to older adults via community care facilities (e.g., DECC, NEC, and other community care centers). These will encompass online platforms, posters and community events, to raise awareness and facilitate participant identification.

### Randomization and allocation

Individual randomization using serially numbered opaque sealed envelopes (SNOSE) will be used to ensure that the allocation sequence is concealed from participants before the group allocation. The PA will prepare about 90 identical, opaque, sealed A5-sized envelopes, with a unique three-digit serial number on the cover of each envelope as an identifier. One-third of the envelopes will contain the action plan for the intervention group, another third the action plan for the audio-based MBI group, and the remaining envelopes the action plan for the waitlist control group. The intervention materials will be inserted into the envelopes, which will be sealed, shuffled, and numbered. Once a participant consents to participate in the study, the research assistant (RA) will open one envelope according to the sequence of the serial number and assign the participant to a treatment condition based on the action plan. Due to the nature of the intervention, no participants will be blinded to the given interventions.

### Blinding

It is not possible to blind the participants to group allocation for psychological interventions, while the assessor who is responsible for the post-test data collection will be blinded. Participants’ personal identifiers will be excluded from the dataset.

### Intervention description

1) **VR-based MBI**

Upon enrolment in the study, participants assigned to the VR-based MBI group will be invited to a briefing session at Lingnan University. The session will take place in a quiet room with suitable lighting and air conditioning. Following the participants’ written informed consent, they will be introduced to the VR-based MBI user manual, VR technology after completing a pre-test survey.

During the briefing session, participants will download the VR-based MBI app developed by our research team and familiarize themselves with wearing the JAPAN JTSK 6th generation upgraded version of 3D virtual reality glasses. They will engage in a 5–10 minute exercise within the VR environment, giving them the opportunity to explore different natural sceneries provided in the mobile app. This exercise aims to acclimate participants to the VR technology and allow them to select environments for their mindfulness practice at home. Participants will be provided with the VR glasses to take home and practice mindfulness for approximately 10–15 minutes daily over the next eight weeks. The app will record their practice time for further analysis.

Weekly telephone follow-ups will be conducted to address any questions or concerns, and reminder messages will be sent once a week. At the end of the eight-week intervention period, participants will complete a post-test questionnaire, with a follow-up test scheduled two months after the intervention.

2) **Audio-based MBI**

Participants in the audio-based group will also attend a briefing session where they will receive information on mindfulness practice. They will listen to the same 10–15 minute audio-based MBI instruction as provided in the VR-based MBI app. Similar to the VR-based MBI group, they will receive reminder messages and telephone follow-ups. Post-test questionnaires will be administered at the end of the intervention, with a follow-up test planned two months later. The only difference between the VR-based MBI and audio-based MBI groups will be the absence of VR technology in the audio-based group.

3) **Usual Care (Waitlist control)**

The third group will be the waitlist control group, who will not attend any briefing session or engage in mindfulness-based interventions during the pilot period. Instead, they will have the opportunity to participate in normal activities provided by community service centers. We will provide the VR-based MBI to the waitlist control group after they have completed the control period.

### The control condition

Fidelity to the intervention protocol will be monitored through regular supervision and adherence checklists. We anticipate potential challenges related to technical issues and user unfamiliarity with virtual reality, but we will address these challenges by providing technical support and conducting orientation sessions and weekly follow-up calls.

### The explanation for the choice of comparators

As mindfulness-based intervention has been proven effective for caregivers of people with dementia, we will involve the components of mindfulness in both the two intervention groups (VR-based MBI vs audio-based MBI), which represent the key primary comparison. Therefore, any extra effects incurred in the intervention group can be attributed to the benefits of using VR. Moreover, the comparison between the two intervention groups and the control group will also demonstrate the effectiveness of media-assisted mindfulness intervention.

### Relevant concomitant care permitted or prohibited during the trial

Relevant concomitant care and interventions have been carefully considered in accordance with the trial’s guidelines. Participants will be permitted to continue any ongoing medications or treatments for chronic pain throughout the study period. However, they will be asked not to initiate any new treatments or therapies during the trial. Participants will be encouraged to maintain their usual daily activities and exercise routines as normal. To preserve the integrity of the intervention, participants will be instructed to refrain from engaging in any mindfulness or meditation practices outside those provided by the study. These measures are intended to enhance the scientific rigor of the trial and ensure that the effects of the mindfulness-based intervention can be accurately evaluated.

### Outcomes

#### Quantitative study.

At the completion of the intervention, the intervention group will be interviewed to evaluate the acceptability and feasibility of VR-based MBI using the Treatment Acceptability/ Adherence Scale with 10 items [21]. The primary outcome is participants’ psychological status at 8 weeks. Two complementary measures will be used to capture multiple dimensions of psychological status, i.e., the Depression, Anxiety, Stress scale (21 items; DASS-21) [22] and the Chinese Version of the Personal Well-being ONS4 (4items; ONS4) [23]. The secondary outcomes will include (a) Cantonese Zarit Burden Scale Short Version [24]; (b) caregivers’ health-related quality of life measured by the EUROHIS-QOL 5-Item Index [25]; (c) caregivers’ mindfulness level measured by the five-facet mindfulness questionnaire [26]; and (d) carer-patient relationship measured by the Relationship Closeness Scale [27].

#### Post-trial qualitative interviews.

Participants of intervention group will be invited to discuss their experiences of MBI via the VR technique, enabling their comments and suggestions to be collected, and the pros and cons of the VR technique discussed.

Qualitative and quantitative findings will be integrated at the interpretation stage using a mixed-methods approach. Themes derived from interview data will be compared with quantitative results to help explain observed outcomes, contextualize adherence and engagement patterns, and inform refinement of the intervention and design of a future definitive trial.

#### Follow-up and incentive.

To minimize the survey effect and to assess the long-term effects of VR-based MBI, a follow-up questionnaire will be administered to assess the study outcomes two months after the completion of the intervention. As the effects of VR-based MBI largely rely on participants’ adherence and compliance, therefore, an attractive incentive is needed.

All participants will be given a HK$ 150 gift voucher after completing the baseline and post-study surveys. A HK$50 gift voucher will be given to participants completing the 2-month follow-up survey, and another HK$50 voucher for completing the qualitative interview.

### Ethical consideration and declarations

#### Confidentiality.

This trial has been approved by the Office of Research and Knowledge Transfer, Lingnan University (Reference No.: EC021–2425) and Institutional Review Board of the University of Hong Kong/ Hospital Authority Hong Kong West Cluster (HKU/HA HKW IRB) (Reference No.: UW24–706). Written informed consent will be sought and obtained from all research participants, and participants have the right to withdraw from the research at any time without penalty. The entire protocol will be uploaded on clinicaltrialregistration.org for public access. The participant-level data and statistical code will be available upon reasonable request to the corresponding author after the completion of the study. Personal information about potential and enrolled participants will be collected, shared, and maintained in strict confidence following the SPIRIT guidelines. The SPIRIT guidelines will be accomplished through a variety of measures, including obtaining informed consent from participants, using unique study identification numbers to de-identify data, storing data securely with restricted access, and only disclosing information to authorized parties with a legitimate need to know. In addition, participants will be informed of their rights to access their personal information and withdraw at any time. Before, during, and after the trial, the participants’ personal information will be protected by these measures.

### Data management

Strict confidentiality will be kept and that the information obtained in the study will be used for research purposes only. The data collected in this study will be kept for 5 years after the study, and personal identifiers will be removed for long term retention of the research data. Participant will not be identified by name in any report of the completed study.

### Data analysis

#### Quantitative data analysis plan.

Quantitative data will be analysed using Stata 17.0. All randomized participants were included in the analysis according to the intention-to-treat (ITT) principle, regardless of their adherence to the assigned intervention or loss to follow-up. Two sensitivity analyses will be conducted: (1) a complete case analysis including only participants with available outcome data; and (2) multiple imputation by chained equations under a missing at random assumption. The imputation model will include treatment allocation, baseline covariates, and variables predictive of missingness and/or the outcome. Twenty imputed datasets will be generated and combined using Rubin’s rules. The plausibility of the missing at random assumption will be explored by comparing baseline characteristics between participants with complete and incomplete data. Results from these sensitivity analyses will be compared with the primary intention-to-treat analysis to assess robustness to different assumptions about missing data.

Continuous variables will be checked for normal distribution. First, descriptive analyses will be conducted. Then, paired sample t-tests will be conducted to examine the level of outcome change between pre-test and post-test. Between-group t-tests will also be calculated to detect differences between the intervention and the control groups. A linear mixed-effects model (LMM) will be employed, accounting for both fixed effects (e.g., intervention group, time, and their interaction) and random effects (e.g., individual variability among participants). Yet, given the anticipated sample size, model adequacy will be evaluated based on convergence and estimate precision. If the model demonstrates instability or overparameterization, a prespecified simplified model will be used (e.g., removal of the interaction term or use of an analysis of covariance at the primary endpoint). These alternatives are intended to preserve valid estimation of the treatment effect while reducing model complexity.

Confounding variables include demographic variables, self-reported physical health, health condition, social support, technology literacy, social service utilisation [28, 29]. Because treatment allocation is randomized, covariate adjustment is not required for unbiased estimation of the treatment effect. Baseline balance will be assessed using standardized mean differences (SMD). Prespecified baseline covariates with SMD > 0.10 will be included as adjustment variables to improve precision, whereas covariates with SMD ≤ 0.10 will not be routinely adjusted for. The primary analysis will remain intention-to-treat, with covariate adjustment used parsimoniously to avoid unnecessary model complexity.

### Qualitative interview—intervention evaluation

After each focus group, digitally recorded audio files will be saved and transcribed by the RA into text. Qualitative data analysis software such as Nvivo will be used for qualitative data analysis. Significant non-verbal and para-linguistic conversations related to participants’ experiences of using VR-based MBI will also be noted and recorded. Thematic analysis will be utilized for data analysis, following six steps of thematic analysis, ensuring familiarity with the data, generating initial codes, searching for themes, reviewing themes, defining and naming themes, and producing the report [30]. The team will use open- coding to identify relevant content in the transcripts and classify all labelled content into several independent themes showing distinct features of the participants’ feedback. Results will then be discussed and consolidated among team members.

### Oversight and monitoring

#### Composition of the data monitoring committee, its role, and reporting structure.

A project team will be formed, including all investigators of universities, institutes and the agencies where the investigators recruited the participants. The team will meet regularly to design the project’s training, intervention, and operation. The team members from the agencies will provide more expert advice on frontline counseling and recruitment.

### Strategies for improving adherence

Weekly telephone follow-ups will be conducted to address any questions or concerns, and reminder messages will be sent once a week. Participants’ daily practice activity (frequency and time) will be recorded in the VR-based mindfulness app. The records will be collected and checked by research assistants via the app Dashboard, allowing the RA to track each participant’s adherence. Possible side-effects will be also checked in and recorded by RA during the telephone follow-ups.

### Process evaluation

We will conduct a thorough process evaluation to assess implementation quality and its impact on outcomes, informing the decision on whether to proceed to a full trial. This evaluation will include multiple dimensions, such as applying the Red-Amber-Green (RAG) framework to evaluate progression criteria [31–33].

### Adverse event reporting and harms

The common adverse effects of using VR include nausea, eye fatigue [34], dizziness, headache, and motion sickness [35]. The fixed background and short practice time will reduce these adverse effects. During the design stage of the VR-based MBI, relevant procedures will be followed to reduce the adverse effects of using VR and to increase participants’ static balance. To manage potential motion sickness, we will prioritize user comfort by gradually exposing participants to VR environments. Additionally, we will optimize the VR experience with stable, natural environments, maintain high frame rates, and provide guidance on minimizing discomfort, including hydration and potential use of ginger supplements or anti-nausea medications.

All participants will be asked at the practice reminders and follow-up if they suffer these side effects. If so, the counsellor will provide counselling on usage of the equipment. If the side effects are severe, the counsellor will ask them to cease use. If a participant reports experiencing emotional difficulty, the research team will also refer them to a specialist or other counsellor.

### Plans for communicating important protocol amendments to relevant parties (e.g., trial participants, ethical committees)

Important protocol modifications (e.g., changes to eligibility criteria, outcomes, and analyses) will be reported to the HKU/HA HKW IRB and Office of Research Knowledge Transfer, Lingnan University.

### Dissemination plans

After the completion of this study, we will disseminate our findings through publication in international, peer-reviewed open-access journals, as well as by presenting them via local media outlets and press conferences. All dissemination outputs will be made publicly available to maximize accessibility for the intended beneficiaries. To facilitate the translation of research evidence into practice, we will seek funding to develop educational resources such as booklets and videos. These materials will be distributed to non-profit organizations that support parents of children with various disabilities. Furthermore, the research team will continue to build on prior academic research and practical collaborations in the field of mindfulness, striving to further refine and enhance mindfulness-based intervention.

### Trial status and timeline

This trial has been registered on clinicaltrials.gov with the ID of NCT07103434. The tentative timeline of the project is presented in Fig 1. Participant recruitment is planned to start in Sep 1^st^, 2025 and completed in Oct 31^st^, 2025. Data collection will be completed within ten months including pretests, posttests and two-month follow-up assessments. Results are expected within three months of data collection completion.

## Discussion

The proposed study aims to explore the feasibility, acceptability and efficacy of a mindfulness-based interventions delivered by VR technology in home settings among family caregivers of people living with dementia. The use of VR in MBIs offers several advantages, including increased engagement, improved attention to instructions, enhanced imagination, and reduced boredom during practice.

The intervention for caregivers of patients with dementia is expected to have positive effects on caregivers’ psychological status, mindfulness and quality of life, care recipients’ quality of life and the relationship between caregiver and care recipient, compared with audio-based MBI and usual care. The findings will shed light on future large-scale intervention trials and implementations to reduce the burden experienced by caregivers of patients of dementia. If found to be acceptable and feasible for caregivers of patients with dementia, this type of intervention can function as an innovative method to be applied to help improve the mental health of caregivers.

The VR method is engaging and flexible, which may enhance participant appeal. Dementia caregivers can easily use this method at any time when they feel they need a moment to take a break from their daily caring tasks. Additionally, VR-based intervention can be combined with other traditional counselling methods as a supplementary intervention for participants with psychological distress [36,37]. By reducing caregiver distress and improving their relationships with care recipients, the healthcare system can potentially experience cost reductions.

Moreover, our study will also call for public and policy attention to care about the mental health of caregivers of patients with dementia. Increased awareness and recognition of the importance of caregiver mental health from various stakeholders, including the public, healthcare professionals, and policymakers, will facilitate the achievement of the desired impact.

While participant adherence to the intervention protocol may pose a potential implementation challenge, the research team will employ active monitoring with regular reminders and encouragement strategies. This structured approach is anticipated to maintain high compliance rates throughout the study duration.

## Conclusion

In conclusion, this study aims to explore the feasibility, acceptability and efficacy among dementia caregivers of the MBI delivered by VR technology (VR-based MBI) in the home environment. If the findings are positive, they could form a foundation for contemplating the integration of VR technology into mindfulness training programs, as well as a promising path for incorporating VR-based mindfulness practices for family caregivers for persons with dementia within the local context of Hong Kong.

## Supporting information

S1 File SPIRIT 2025 editable checklist. (DOCX)

S2 File Research Protocol_V1. (PDF)
