# Supplementary material for: A feasibility study on the efficacy of a VR-based mindfulness intervention for dementia caregivers in the home environment: A pilot randomized control trial protocol
Source: PLoS One. 2026 Apr 29;21(4):e0347988. doi: 10.1371/journal.pone.0347988 (PMC13127938; doi:10.1371/journal.pone.0347988)
Supplement: S2 File — (PDF) [file pone.0347988.s002.pdf]

Study Protocol (Version 1; Nov 4, 2024)

Title:
A feasibility study on the efficacy of a VR-based mindfulness intervention for dementia caregivers in the home environment: A pilot randomized control trial

Site PI: Prof Doris Yu, School of Nursing, The University of Hong Kong
Lead PI: Dr Cheng SHI, School of Graduate Studies, Lingnan University

a. **Justification for Conducting the Project:**

(i) **Health needs of the local community**

As the global population ages, the growing prevalence of dementia has become a worldwide public health concern (1). Dementia, a progressive disorder, leads to deteriorating perceptual and cognitive abilities, behavioral changes, and loss of independently (2). Dementia impairs patients' social or occupational functioning and is a major cause of disability in older age. These symptoms not only impact the quality of life of people with dementia but also cause psychological distress for their caregivers (2,3). Currently, 50 million people live with dementia worldwide, which is predicted to rise to 82 million by 2030(4). An ongoing modelling study (5), which is part of a larger project named, "Tools to Inform Policy: Chinese Communities' Action in Response to Dementia (TIP-CARD)" shows that 178,139 people aged 60 years and older live with dementia in Hong Kong in 2021, and over 70% of dementia costs relate to informal care.

Dementia care largely rely on informal caregivers, such as family members, friends, and neighbors. These caregivers are vital partners in the health system and contribute to the realisation of healthy ageing and aging-in-place initiatives. In Hong Kong, over 62.6% of people with dementia have at least two informal caregivers (5). With input from multiple informal carers, the average informal care time amounts to 400 hours per month, 40% of which is attributed to managing behavioral and psychological symptoms of dementia and ensuring their safety (supervision time) (5).

Informal caregivers' physical and mental health are adversely affected by the demanding caregiving tasks they perform, leading to a higher level of psychological distress (6). The changing behaviors and functional dependence of people living with dementia require informal caregivers to spend longer hours and better cognitive preparedness for taking care of the patients, which may lead to a higher level of psychological distress (7). In Hong Kong, around 15.5% of dementia caregivers experienced burnout (6). Especially during the COVID-19 pandemic, 64.7% of people caring for someone with dementia reported suffering from probable depression and increased levels of anxiety and stress (8). The Chief Executive's 2022 Policy Address highlights enhancing support for caregivers and enhancing their mental well-being. However, very limited support is currently available for caregivers of people with dementia in the local community.

Considering the aforementioned features and impacts of caring for individuals with dementia, there is an urgent need for a self-instructed, flexible, and convenient intervention within the home environment to effectively enhance caregivers' psychological well-being in Hong Kong.

(ii) What is the existing evidence before this project based on an up-to-date literature search?

Evidence on interventions supporting dementia caregivers is limited and mainly restricted to Western countries. A recent large-scale systematic review on the effectiveness of dementia-related interventions in Chinese communities found very limited evidence about dementia caregivers among 525 included unique randomized control trials (RCTs) (9). Among the existing evidence, different types of interventions to reduce caregivers' stress and improve their psychological well-being have been studied, including case management, cognitive stimulation, cognitive and behavioral interventions, education, physical activity interventions, psychosocial support, relaxation and skill-building (10). Although most psychosocial interventions appear to be effective at reducing caregiver stress and psychological morbidity, treatment effect sizes have been small and inconsistent. Among these interventions, mindfulness-based interventions (MBIs) have been widely used to reduce psychological distress in dementia caregivers and show consistent and promising results (2).

Mindfulness refers to "the awareness that emerges through paying attention on purpose, in the present moment, and non-judgmentally to the unfolding of experience moment by moment" (11). Several recent systematic reviews provide robust evidence for the medium to large effect of MBIs on reducing caregivers' psychological distress, depression, burden, and enhancing quality of life by improving their ability to cater to the needs of people with dementia and managing daily stress and emotional challenges associated with caregiving (2,12), as well as enhancing relationship between caregivers and recipients (2).

Dementia caregivers bear significant daily caregiving responsibilities and dedicate the majority of their time to companionship and ensuring the safety of people with dementia. This responsibility often leaves caregivers with limited time and availability to engage in mindfulness practice outside of the home. However, the practice of mindfulness requires conscious effort and can be difficult to maintain in the home environment, especially for novice meditators who are already expending cognitive resources to control their self-regulatory skills (13). Beginning mindfulness practitioners commonly report easily being distracted during practice, fall asleep, wrestle with tiredness or ambivalence, difficulty of incorporating mindfulness practice into their daily life (14). The adoption of digital technology may support beginners' mindfulness practice, such as Web-based interventions (15) or smartphone apps (16) that can deliver guided audio-recorded mindfulness practice in facing the challenges of practicing and adhering to mindfulness (17). However, there are limitations with these audio-based MBIs , including a low adherence rate and the failure of participants to maintain these practices over the long term (17).

(iii) How will this project add value to existing evidence in addressing the health needs of the local community?

Virtual reality (VR) is an immersive technology that may boost the advantages of mindfulness meditation and facilitate participants' attention management. It refers to the use of a headset to create a 360-degree virtual environment, incorporating realistic 3D graphics, stereoscopic viewing, and head tracking (18).The use of VR technology with natural environments embedded in the system to assist mindfulness practice is supported by attention restoration theory (ART) (19). Natural stimuli in VR have the potential to reduce mental fatigue and increase attention (20). Additionally, nature can enhance ecological behavior, both in real-world and virtual experiences (21). The immersive nature of VR blocks distractions, allowing participants to focus solely on mindfulness practice (18).

VR-based mindfulness practice in a pleasant and immersive virtual environment may relieve physical and mental discomforts frequently experienced by beginning mindfulness practitioners without compromising their present-moment awareness (20). It also provides access to physically or cognitively inaccessible stimuli, enhancing guided imaginary mindfulness (21). Furthermore, VR also facilitates participants' application of mindfulness skills to real-life practice, and its non-traditional format may further increase their acceptance of the therapy (20).

VR-based MBI has recently been proposed as a medium to support mindfulness practice and become an effective way to improve participants' psychological well-being and cultivate spiritual experiences, especially strong feelings of awe (22). Specifically, compared to audio-based mindfulness, VR-based MBIs have been shown to offer several additional benefits, including improved well-being (23), higher treatment adherence (24), reductions in vigor, fatigue, and confusion (25) and more enjoyable experiences (26). However, VR-based mindfulness practice studies are predominantly conducted in a clinic environment or specific settings rather than a home environment (20). Practicing in home environment may enable participants to feel safe and remove their concerns about leaving the person with dementia. VR-based MBI practice can be time- and cost-efficient, simple to initiate and complete, and practical for caregivers at home.

Meanwhile, local evidence on VR-based interventions targeting psychological health issues such as depression, anxiety, and stress (27), alongside studies on the benefits of mindfulness for dementia caregivers' well-being (28) provides form a foundation for contemplating the integration of VR technology into mindfulness training programs. This collective body of research suggests a promising path for incorporating VR-based mindfulness practices for family caregivers for persons with dementia within the local context of Hong Kong.

b. **Aim and Objectives:**

This pilot study aims to explore the feasibility, acceptability and efficacy among dementia caregivers of the MBI delivered by VR technology (VR-based MBI) in the home environment. The specific research questions and hypotheses are as follows:

- **How do dementia caregivers perceive VR-based MBI?**

We will explore the feasibility, acceptability and understanding of using VR technology or audio to learn MBI among caregivers of people with dementia through a focus group qualitative study and a quantitative study using the Treatment Acceptability/adherence Scale. The readiness of caregivers to practice MBI at home after observing group sessions will also be explored.

- **Does the VR-based MBI offer additional benefits to psychological well-being compared with audio-based MBI and usual care?**

We will address this question through a 3-arm pilot randomized controlled rails (RCT). Using an 8-week audio-based MBI protocol and usual care as comparing conditions. We hypothesize that among caregivers of people with dementia:

HP1: The VR-based MBI group will show greater improvement in psychological well-being (**Primary Outcome**) than the audio-based MBI group, while the audio-based MBI group will show greater improvement than waitlist control group

Psychological well-being will be assessed through depression, anxiety and stress, as well as general wellbeing.

- **Does the VR-based MBI offer additional benefits to caregivers' mindfulness and quality of life, care recipients' quality of life and the relationship between caregiver and care recipient, compared with audio-based MBI and usual care?**

We will address this question through the RCT mentioned above. Compared with the audio-based MBI and usual care groups, we hypothesize that:

HP2: The VR-based MBI group will show greater improvements in caregiver burden, mindfulness level, and quality of life (**Secondary Outcomes**) than the audio-based MBI group, while the audio-based MBI group will show greater improvement than waitlist control group;

HP3: The relationship between VR-based MBI group caregivers and the care recipients (**Secondary Outcomes**) will show greater improvement than the audio-based MBI group, while the audio-based MBI group will show greater improvement than waitlist control group;

The proposed study will adopt the RE-AIM framework to plan interventions that had the potential to: (1) reach a high proportion of dementia caregivers, (2) effectively increase caregivers' psychological well-being and quality of life; (3) be scalable to improve potential adoption across care settings, (4) be implemented at a reasonable cost, and (5) lead to psychological well-being maintenance and be sustained in typical community settings.

c. **Project Plan:**

(i) Target group

<u>Participants</u>

Caregivers of persons with mild-to-moderate dementia.

<u>Inclusion criteria:</u>

- Aged 18 years or older;
- Primary informal caregivers who spend over four hours/day taking care of a community-dwelling person who has been clinically diagnosed with any type of mild-to-moderate dementia at least 6 months;
- Self-reported psychological distress
- Able to speak and read Cantonese.

Exclusion criteria:

- Have practiced mindfulness-based intervention for at least 3 months in the past;
- Have hearing or visual impairment that cannot practice mindfulness via wearing VR glasses embedded in a mobile phone app;
- Do not own a smart phone that can install the VR-based MBI app;
- Caring for a person with dementia living in a residential care setting;
- Caring a person with severe level of dementia;
- Have been diagnosed with any mental disorder or on-site of psychotic disorders;
- Receiving any other type of mental health intervention;
- Participants with a history of motion sickness (due to the risk of VR triggering motion sickness)
- Unable to understand Cantonese.

We propose a sample size of 90 participants for our three-arm RCT pilot study, with 30 participants in each arm, to ensure adequate distribution and feasibility while minimizing costs. This size allows us to gather preliminary data and insights into trial logistics, as well as sufficient qualitative feedback through focus group discussions. Additionally, this sample size supports our ability to estimate potential effect sizes (from 0.4 to 0.54) identified from previous studies (22,24) , refine hypotheses, and inform the design of a larger trial.

Given that over 178,139 residents live with dementia in Hong Kong (5), we anticipate that it will be feasible to identify and recruit 90 older adults with dementia and their respective caregivers. To improve **Reach**, we will leverage our established network comprising local dementia care facilities, support groups, and community organizations. Building upon prior engagements with dementia caregivers, such as past participants, we aim to employ targeted outreach methods. These will encompass online platforms, posters and community events, to raise awareness and facilitate participant identification.

(ii) Implementation plan

Design

This proposed study will be a pilot RCT to examine the feasibility, acceptability and potential effectiveness of implementing the intervention with caregivers of people with dementia. Ethics approval will be obtained from the Office of Research and Knowledge Transfer, Lingnan University and HKU/HA HKW Institutional Review Board (HKU/HA HKW IRB).

Setting

Participants will practice MBI at their home. For enhancing the **adoption**, we will collaborate with local dementia care facilities and support groups and engage key stakeholders by highlighting the potential benefits of the intervention and demonstrate the feasibility and relevance of the intervention. Regular communication will be conducted to ensure a smooth adoption process.

Specifically, participants will be recruited through (a) three District Elderly Community Centers (DECCs) participating in the Dementia Community Support Scheme (DCSS), (b) participants of a previous dementia study, and (c) open recruitment from community service centers and other health and social care facilities.

First, three DECCs in the Tuen Mun area, who are DCSS partners, will serve as the main recruitment sites for the proposed. The Hong Kong SAR Government has launched the DCSS to provide multi-disciplinary community support services through medical-social collaboration for people with mild or moderate dementia and family carers since 2017 (The Government of the Hong Kong SAR, 2018, July 4). Apart from DCSS, the three DCSS-partner DECCs also provide other support and services for caregivers of people with mild-to-moderate dementia. Currently, around 330 caregivers of people with dementia are registered service users.

Second, we will contact participants (i.e., dementia caregiver) of our previous TIP-CARD study. In TIP-CARD project, 750 participants of the survey in 2021-2022 have already consented to being contacted for other dementia-related projects.

Third, when necessary, we will also conduct open recruitment. We will distribute our recruitment poster to older adults via community care facilities (e.g., DECC, NEC, and other community care centres) and mass email to participants in a community service project (n>500) for which the PA serves as project manager.

<u>Procedures</u>

The pilot RCT is a 3-arm, parallel, single-blinded trial to examine the feasibility, acceptability and potential effectiveness of the VR-based MBI, following the “Consolidated Standards of Reporting Trials” (CONSORT) guidelines(29). The intervention will last eight weeks with assessment periods at baseline, posttreatment, and 2-months follow-up. We will also conduct focus group for the intervention group after the intervention and at follow-up, using an interview guide. A consort diagram of procedures for our RCT pilot design and RE-AIM considerations is shown in Figure 1.

Figure 1. Consort diagram of procedures for randomised controlled trail design and RE-AIM considerations

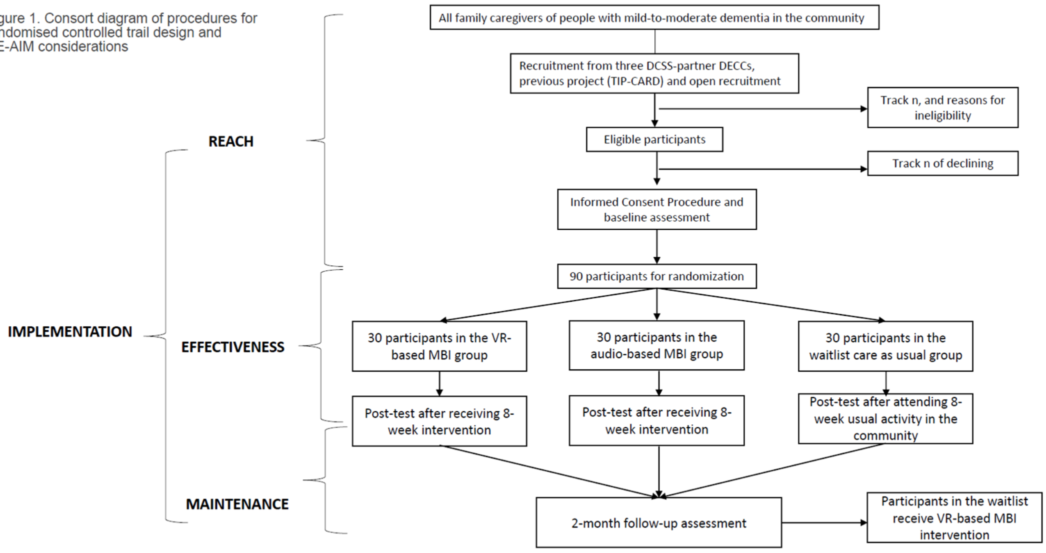

<u>Randomisation</u>

Eligible participants will be randomly assigned into three groups: A) VR-based MBI (Intervention group) B) audio-based MBI group (control group) and C) Usual care group (Waitlist control group). Individual randomization using serially numbered opaque sealed envelopes (SNOSE) will be used to ensure that the allocation sequence is concealed from participants before the group allocation. The PA will prepare about 90 identical, opaque, sealed A5-sized envelopes, with a unique three-digit serial number on the cover of each envelope as an identifier. One-third of the envelopes will contain the action plan for the intervention group, another third the action plan for the audio-based MBI group, and the remaining envelopes the action plan for the waitlist control group. The intervention materials will be inserted into the envelopes, which will be sealed, shuffled, and numbered. Once a participant consents to participate in the study, the RA will open one envelope according to the sequence of the serial number and assign the participant to a treatment condition based on the action plan. No participants will be blinded to the given interventions.

<u>Intervention and control groups</u>

A) VR-based MBI group （Intervention group）

Upon enrolment in the study, participants assigned to the VR-based MBI group will be invited to a briefing session at Lingnan University. The session will take place in a quiet room with suitable lighting and air conditioning. Following the participants' written informed consent, they will be introduced to the VR-based MBI user manual, VR technology after completing a pre-test survey.

During the briefing session, participants will download the VR-based MBI app developed by our research team and familiarize themselves with wearing the JAPAN JTSK 6th generation upgraded version of 3D virtual reality glasses. They will engage in a 5-10 minute exercise within the VR environment, giving them the opportunity to explore different natural sceneries provided

in the app. This exercise aims to acclimate participants to the VR technology and allow them to select environments for their mindfulness practice at home. Participants will be provided with the VR glasses to take home and practice mindfulness for approximately 10-15 minutes daily over the next eight weeks. The app will record their practice time for further analysis. Weekly telephone follow-ups will be conducted to address any questions or concerns, and reminder messages will be sent once a week.

B) Audio-based MBI group (Control group)

Participants in the audio-based group will also attend a briefing session where they will receive information on mindfulness practice. They will listen to the same 10-15 minute audio-based MBI instruction as provided in the VR-based MBI app. Similar to the VR-based MBI group, they will receive reminder messages and telephone follow-ups. The only difference between the VR-based MBI and audio-based MBI groups will be the absence of VR technology in the audio-based group.

C) Usual care group (Waitlist control group)

The third group will be the waitlist control group, who will not attend any briefing session or engage in mindfulness-based interventions during the pilot period. Instead, they will receive usual care and services provided by community service centres. No additional or new intervention will be provided to them during the study period. We will provide the VR-based MBI to the waitlist control group participants after they have completed the control period.

Blinding

It is not possible to blind the participants to group allocation for psychological interventions, while the RA who is responsible for the post-test data collection will be blinded. Participants' personal identifiers will be excluded from the dataset.

Assessment

Timeline of overall evaluation plan on intervention effectiveness is shown in Figure 2. All participants will complete a 30-minute face-to-face survey comprising a set of assessment on psychological stress, well-being, and other relevant outcomes before the intervention and after the 8-week intervention. To minimize the survey effect and to assess the long-term effects, we will also conduct a phone interview as a follow-up survey at two months after the intervention.

The VR-based MBI group will also participate in two face-to-face, 60-minute, audio-recorded focus group discussions—one immediately after the intervention and another at the two-month follow-up—to provide in-depth feedback on their experience with the intervention. These discussions will help us understand the impact of the VR-based MBI and identify any potential benefits or challenges.

All face-to-face surveys and focus groups will be organised and taken place at Lingnan University.

Figure 2 Evaluation timeline

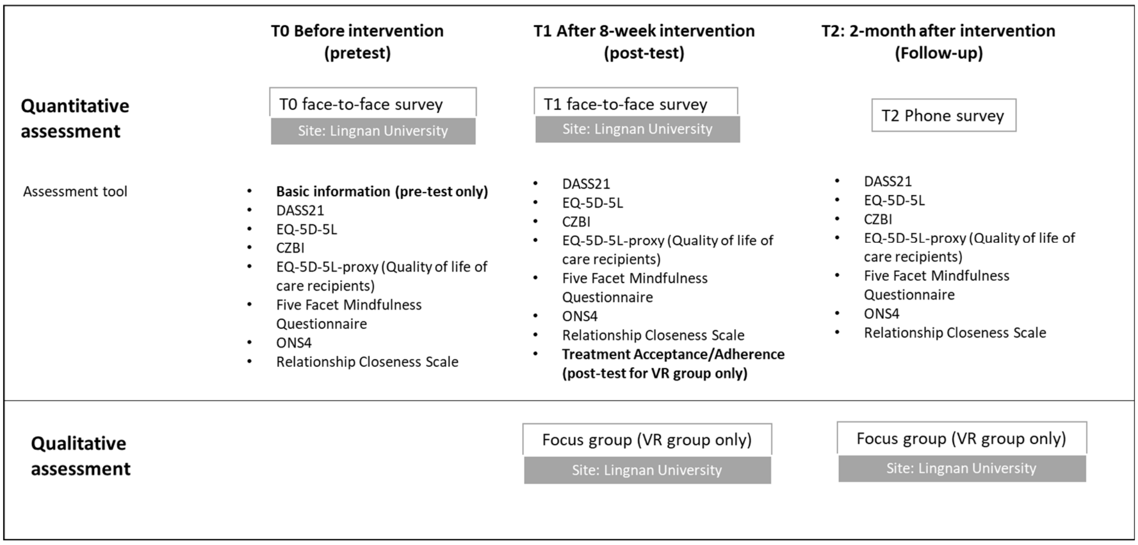

Incentives for participants

For your participation, you will receive a HK$100 supermarket coupon after completing pre and post-test and an additional HK$50 coupon after completing a follow-up phone survey at two months after intervention. If you are in the VR-based MBI group and participate in the focus group discussions, you will be given an additional HK$50 supermarket coupon for each session you attend.

(iii) Contingency/alternative plan if any problem encountered during implementation

Possible adverse effects of using VR

The common adverse effects of using VR include nausea, eye fatigue (41), dizziness, headache, and motion sickness.(30) The fixed background and short practice time will reduce these adverse effects. During the design stage of the VR-based MBI, relevant procedures will be followed to reduce the adverse effects of using VR and to increase participants' static balance. To manage potential motion sickness, we will prioritize user comfort by gradually exposing participants to VR environments. Additionally, we will optimize the VR experience with stable, natural environments, maintain high frame rates, and provide guidance on minimizing discomfort, including hydration and potential use of ginger supplements or anti-nausea medications.

All participants will be asked at the practice reminders and follow-up if they suffer these side effects. If so, the counsellor will provide counselling on usage of the equipment. If the side effects are severe, the counsellor will ask them to cease use. If a participant reports experiencing emotional difficulty, the research team will also refer them to a specialist or other counsellor.

(iv) Cross-sector collaboration

A project team will be formed including investigators of Lingnan University (LU）, Chinese University of Hong Kong (CUHK), The University of Hong Kong (HKU) collaborating with

service providers from three DECCs, i.e., Yan Oi Tong, The Neighbourhood Advice-Action Council, Caritas District Elderly Centre-Yuen Long, and Pok Oi Hospital Mrs. Wong Tung Yuen District Elderly Community Centre. The team will meet regularly to design the training, intervention, and operation of the project.

(v) Indicators and targets

- **Reach**

Reach indicators encompass the number of dementia caregivers who express interest in participating and those who actually engage in the program, and the representativeness of participants considering their demographic characteristics.

Qualitative feedback on barriers and facilitators affecting recruitment will be gathered from participants and local stakeholders.

- **Effectiveness**

Overall evaluation plan is shown in Figure 2.

Evaluation of the feasibility and acceptance of VR-based MBI

(Quantitative indicator) At the completion of the intervention, the intervention group will be interviewed to evaluate the acceptability and feasibility of VR-based MBI using the Treatment Acceptability/ Adherence Scale with 10 items (31) .

(Qualitative indicator) Participants will be invited to discuss their experiences of MBI via the VR technique, enabling their comments and suggestions to be collected, and the pros and cons of the VR technique discussed.

Evaluation of efficacy of VR-based MBI

(Quantitative indicators) The primary outcomes will be participants' psychological well-being (measured by the Depression, Anxiety, Stress scale (21 items) (32) and the Chinese Version of the Personal Well-being ONS4 (4items) (33). The secondary outcomes will include (a) Cantonese Zarit Burden Scale Short Version (34); (b) caregivers' health-related quality of life measured by the EUROHIS-QOL 5-Item Index (35); (c) caregivers' mindfulness level measured by the five-facet mindfulness questionnaire (36) ; and (d) carer-patient relationship measured by the Relationship Closeness Scale (37).

- **Adoption**

At the setting level, quantitative adoption indicators will encompass the percentage of participants from various recruitment channels, reasons for setting exclusions, participation rates of approached settings, and a comparison of participating settings with non-participants based on characteristics.

At the staff levels, adoption evaluation will involve gathering qualitative feedback on intervention delivery, such as reasons driving adoption or non-adoption of the intervention, and how the intervention could be integrated into their existing practices and services, along with their feedback on the potential for broader adoption within their organisation or beyond.

For caregivers, qualitative methods will be employed to assess their participation and experiences during the intervention period.

- **Implementation**

Implementation will be evaluated by monitoring the percentage of intervention sessions that are delivered as intended, any adaptations made to the intervention during the study, any additional costs incurred, and the consistency of intervention delivery across different study arms.
We will also collect qualitative feedback on the barriers and facilitators encountered during the implementation process and the overall challenges faced by participants and staff.

- **Maintenance**

Maintenance will be assessed by tracking the retention rate of participants who continue to engage with the VR-based mindfulness intervention beyond the initial trial period. We will conduct a 2-month follow-up assessment to evaluate whether the benefits observed during the study are sustained over time. Additionally, we will gather qualitative feedback from multiple stakeholders to assess the intervention's integration into existing practice and the support required to maintain intervention effectiveness.

(vi) Evaluation plan (RE-AIM framework)

- **Reach of the target population**

During the recruitment and implementation, we will track the number of dementia caregivers who show interest in the program and those who proceed to participate. We will also collect demographic data from participants to assess how well they represent the target population of dementia caregivers in Hong Kong. Additionally, we will gather qualitative data through weekly call, focus groups and stakeholder interviews to understand the factors that influence recruitment and participation.

- **Effectiveness (or Efficacy)**

<u>Evaluation of the feasibility and acceptance of VR-based MBI</u>

After completing the 8-week intervention, the VR-based MBI group will participate a survey to evaluate the acceptability and feasibility. Meanwhile, a longitudinal **qualitative** element of the study will be included by undertaking face-to-face focus group discussions with participants in the VR-based MBI group at the complication of the intervention and at 2-months follow-up. It is envisaged there will be 3-4 focus groups, each including 5-6 participants, at each timepoint. The PA and Co-As will facilitate the focus group discussions employing a semi-structured interview method will be adopted. The group discussions will be audio-recorded.

<u>Evaluation of efficacy of VR-based MBI</u>

Participants will be invited to complete a face-to-face survey before and after the intervention to assess efficacy in terms of target outcomes. At 2-months follow-up, all three groups will be contacted for a telephone survey using the same assessment tools.

- **Adoption by target staff, settings, systems and communities**

We will track relevant participants information for calculating the indicators after recruitment. We will also collect caregivers' feedback on the adoption of the intervention through weekly phone calls and the focus group discussions after the intervention. During the recruitment and after the intervention period, we will also gather staff opinions on how this intervention can be integrated into their existing care practices and services and on the potential for wider adoption of the intervention within their organisation or across other institutions.

- **Implementation: consistency, costs and adaptions made during delivery**

We will track whether the intervention is being delivered as intended and any necessary adaptations made during the study. We will collect feedback from weekly call and focus group discussions after the intervention and exercise time records from the app.

- **Maintenance/sustainment of intervention effects in individuals and settings over time**

The long-term maintenance of the intervention's effects will be evaluated through follow-up assessments with participants and feedback from staff and settings that have adopted the VR-based MBI.

(vii) **Results analysis**

Quantitative data analysis plan

Quantitative data will be analysed using STATA 17.0. By intention-to-treat analysis, participants who are lost or refuse the follow-up will be treated as "no change in psychological well-being". Continuous variables will be checked for normal distribution. First, descriptive analyses will be conducted. Then, paired sample t-tests will be conducted to examine the level of outcome change between pre-test and post-test. Between-group t-tests will also be calculated to detect differences between the intervention and the control groups. A linear mixed-effects model (LMM) will be employed, accounting for both fixed effects (e.g., intervention group, time, and their interaction) and random effects (e.g., individual variability among participants). Confounding variables include demographic variables, self-reported physical health, health condition, social support, technology literacy, social service utilisation (38,39).

Qualitative interview—intervention evaluation

After each focus group, digitally recorded audio files will be saved and transcribed by the RA into text. Nvivo 11 will be used for qualitative data analysis. Significant non-verbal and para-linguistic conversations related to participants' experiences of using VR-based MBI will also be noted and recorded. Thematic analysis will be utilized for data analysis, following six steps of thematic analysis, ensuring familiarity with the data, generating initial codes, searching for themes,

reviewing themes, defining and naming themes, and producing the report (40). The PA and RA will use open- coding to identify relevant content in the transcripts. Then the PA will classify all labelled content into several independent themes showing distinct features of the participants' feedback. Results will then be discussed and consolidated in panel meetings with other CoAs.

Ethics consideration

Major ethnical issues

The study follows the Declaration of Helsinki on medical protocol and ethics. Ethics approval will be obtained from the HKU/HA HKW Institutional Review Board (HKU/HA HKW IRB). Participants are voluntary to join the study. A written informed consent, which will include the research title, purpose, explanation of the research, and the procedures of the study, will be obtained from each eligible participant. Risks and benefits are also explained clearly to the participants. Participants have the right to withdraw from the study at any time. They will be protected from discomfort and harm during the study. Further, anonymity and confidentiality of the participants will be strictly protected.

Data Protection

Strict confidentiality will be kept and that the information obtained in the study will be used for research purposes only. The data collected in this study will be kept for 5 years after the study, and personal identifiers will be removed for long term retention of the research data. Participant will not be identified by name in any report of the completed study.

Funding Source

This study will be funded by the Health and Medical Research Fund (HMRF).

d. **Key References:**

1. Nichols E, Steinmetz JD, Vollset SE, Fukutaki K, Chalek J, Abd-Allah F, et al. Estimation of the global prevalence of dementia in 2019 and forecasted prevalence in 2050: an analysis for the Global Burden of Disease Study 2019. The Lancet Public Health. 2022;7(2):e105–25.

2. Shim M, Tilley JL, Im S, Price K, Gonzalez A. A systematic review of mindfulness-based interventions for patients with mild cognitive impairment or dementia and caregivers. Journal of Geriatric Psychiatry and Neurology. 2021;34(6):528–54.

3. Millán-Calenti JC, Lorenzo-López L, Alonso-Búa B, Labra C, González-Abraldes I, Maseda A. Optimal nonpharmacological management of agitation in Alzheimer's disease: challenges and solutions. Clin Interv Aging Published online. 2016;175–84.

4. World Health Organisation. WHO fact sheet on dementia [Internet]. 2023 [cited 2024 Mar 13]. Available from: https://www.who.int/news-room/fact-sheets/detail/dementia

5. Shi C. Cost of dementia in Hong Kong [Internet]. Symposium: Hong Kong and Global Responses to Dementia; 2023 Feb 18. Available from: https://www.tip-card.hku.hk/online-symposium-2023

6. Chan CY, Cheung G, Martinez-Ruiz A, Chau PY, Wang K, Yeoh EK, et al. Caregiving burnout of community-dwelling people with dementia in Hong Kong and New Zealand: a cross-sectional study. BMC geriatrics. 2021;21:1–15.

7. Allen AP, Buckley MM, Cryan JF, Ní Chorcoráin A, Dinan TG, Kearney PM, et al. Informal caregiving for dementia patients: the contribution of patient characteristics and behaviours to caregiver burden. Age and ageing. 2020;49(1):52–6.

8. Fong TKH, Cheung T, Chan WC, Cheng CPW. Depression, Anxiety and Stress on Caregivers of Persons with Dementia (CGPWD) in Hong Kong amid COVID-19 Pandemic. International Journal of Environmental Research and Public Health. 2022 Jan;19(1):184.

9. Shi C, Chen S, Salcher-Konrad M, Choy JC, Luo H, Leung DKY, et al. Effectiveness of interventions for people living with dementia and their carers in Chinese communities: protocol for a systematic review and meta-analysis of randomised controlled trials. BMJ open. 2021;11(8):e047560.

10. Huggins M, Pesut B, Puurveen G. Interventions for Caregivers of Older Adults with Dementia Living in the Community: A Rapid Review of Reviews. Can J Aging. 2023 Feb 17;1–9.

11. Bishop SR, Lau M, Shapiro S, Carlson L, Anderson ND, Carmody J, et al. Mindfulness: A proposed operational definition. Clinical psychology: Science and practice. 2004;11(3):230.

12. Collins RN, Kishita N. The effectiveness of mindfulness-and acceptance-based interventions for informal caregivers of people with dementia: A meta-analysis. The Gerontologist. 2019;59(4):e363–79.

13. Lymeus F, Lundgren T, Hartig T. Attentional effort of beginning mindfulness training is offset with practice directed toward images of natural scenery. Environment and Behavior. 2017;49(5):536–59.

14. Lomas T, Cartwright T, Edginton T, Ridge D. A qualitative analysis of experiential challenges associated with meditation practice. Mindfulness. 2015;6:848–60.

15. Winter N, Russell L, Ugalde A, White V, Livingston P. Engagement strategies to improve adherence and retention in web-based mindfulness programs: systematic review. Journal of Medical Internet Research. 2022;24(1):e30026.

16. Linardon J. Can acceptance, mindfulness, and self-compassion be learned by smartphone apps? A systematic and meta-analytic review of randomized controlled trials. Behavior Therapy. 2020;51(4):646–58.

17. Economides M, Martman J, Bell MJ, Sanderson B. Improvements in stress, affect, and irritability following brief use of a mindfulness-based smartphone app: a randomized controlled trial. Mindfulness. 2018;9(5):1584–93.

18. Navarro-Haro MV, López-del-Hoyo Y, Campos D, Linehan MM, Hoffman HG, García-Palacios A, et al. Meditation experts try Virtual Reality Mindfulness: A pilot study evaluation of the feasibility and acceptability of Virtual Reality to facilitate mindfulness practice in people attending a Mindfulness conference. Ito E, editor. PLoS ONE. 2017 Nov 22;12(11):e0187777.

19. Ohly H, White MP, Wheeler BW, Bethel A, Ukoumunne OC, Nikolaou V, et al. Attention Restoration Theory: A systematic review of the attention restoration potential of exposure to natural environments. Journal of Toxicology and Environmental Health, Part B. 2016;19(7):305–43.

20. Failla C, Marino F, Bernardelli L, Gaggioli A, Doria G, Chilà P, et al. Mediating Mindfulness-Based Interventions with Virtual Reality in Non-Clinical Populations: The State-of-the-Art. Healthcare. 2022 Jun 29;10(7):1220.

21. Deringer SA, Hanley A. Virtual reality of nature can be as effective as actual nature in promoting ecological behavior. Ecopsychology. 2021;13(3):219–26.

22. Ma J, Zhao D, Xu N, Yang J. The effectiveness of immersive virtual reality (VR) based mindfulness training on improvement mental-health in adults: A narrative systematic review. EXPLORE. 2022 Aug;S1550830722001227.

23. Cawley A, Tejeiro R. Brief Virtual Reality Mindfulness is More Effective than Audio Mindfulness and Colouring in Reducing Stress in University Students. Mindfulness. 2024;15:272–81.

24. Navarro-Haro MV, Modrego-Alarcón M, Hoffman HG, López-Montoyo A, Navarro-Gil M, Montero-Marin J, et al. Evaluation of a Mindfulness-Based Intervention With and Without Virtual Reality Dialectical Behavior Therapy® Mindfulness Skills Training for the Treatment of Generalized Anxiety Disorder in Primary Care: A Pilot Study. Front Psychol. 2019 Jan 28;10:55.

25. Tarrant J, Jackson R, Viczko J. A Feasibility Test of a Brief Mobile Virtual Reality Meditation for Frontline Healthcare Workers in a Hospital Setting. Front Virtual Real. 2022 Jan 26;3:764745.

26. Gentile DA, Kim EL. Comparing Virtual Reality, Video, and Audio-Guided Meditations in Fostering Positive Attitudes toward Meditation. Mindfulness. 2024 Mar;15(3):586–600.

27. Tan YL, Chang VYX, Ang WHD, Ang WW, Lau Y. Virtual reality exposure therapy for social anxiety disorders: a meta-analysis and meta-regression of randomized controlled trials. ANXIETY STRESS AND COPING. 2-4 PARK SQUARE, MILTON PARK, ABINGDON OR14 4RN, OXON, ENGLAND: TAYLOR & FRANCIS LTD; 2024.

28. Kor PPK, Liu JYW, Chien WT. Effects of a Modified Mindfulness-Based Cognitive Therapy for Family Caregivers of People With Dementia: A Randomized Clinical Trial. Vol. 61, GERONTOLOGIST. JOURNALS DEPT, 2001 EVANS RD, CARY, NC 27513 USA: OXFORD UNIV PRESS INC; 2021. p. 977–90.

29. Schulz KF, Altman DG, Moher D. CONSORT 2010 statement: updated guidelines for reporting parallel group randomised trials. Journal of Pharmacology and pharmacotherapeutics. 2010;1(2):100–7.

30. Park S, Lee G. Full-immersion virtual reality: Adverse effects related to static balance. Neuroscience letters. 2020;733:134974.

31. Milosevic I, Levy HC, Alcolado GM, Radomsky AS. The Treatment Acceptability/Adherence Scale: Moving Beyond the Assessment of Treatment Effectiveness. Cogn Behav Ther. 2015;44(6):456–69.

32. Wang K, Shi HS, Geng FL, Zou LQ, Tan SP, Wang Y, et al. Cross-cultural validation of the depression anxiety stress scale–21 in China. Psychological assessment. 2016;28(5):e88.

33. Benson T, Sladen J, Liles A, Potts HW. Personal Wellbeing Score (PWS)—a short version of ONS4: development and validation in social prescribing. BMJ open quality. 2019;8(2):e000394.

34. Tang JY man, Ho AH yan, Luo H, Wong GH yan, Lau BH po, Lum TY sang, et al. Validating a Cantonese short version of the Zarit Burden Interview (CZBI-Short) for dementia caregivers. Aging & Mental Health. 2016;20(9):996–1001.

35. Wong EL yi, Cheung AW ling, Wong AY kwan, Xu RH, Ramos-Goñi JM, Rivero-Arias O. Normative profile of health-related quality of life for Hong Kong general population using preference-based instrument EQ-5D-5L. Value in Health. 2019;22(8):916–24.

36. Baer RA, Smith GT, Lykins E. Construct validity of the five facet mindfulness questionnaire in meditating and nonmeditating samples. Assessment. 2008;15(3):329–42.

37. Dibble JL, Levine TR, Park HS. The Unidimensional Relationship Closeness Scale (URCS): Reliability and validity evidence for a new measure of relationship closeness. Psychological Assessment. 2012;24(3):565–72.

38. Buckingham S, Tu G, Elliott L, Poole R, Walker T, Bland E, et al. Digital competence and psychological wellbeing in a social housing community: a repeated survey study. BMC Public Health. 2023;23(1):2002.

39. Choi YJ. Service Availability, Service Use, and Psychological Well-Being of Older Residents of Residential Care Facilities and Nursing Homes. Seniors Hous Care J. 2019;27(1):51–61.

40. Kiger ME, Varpio L. Thematic analysis of qualitative data: AMEE Guide No. 131. Medical Teacher. 2020 Aug 2;42(8):846–54.
